# Pediatric catatonia: A case report and review of the management

**DOI:** 10.1002/pcn5.70329

**Published:** 2026-04-12

**Authors:** Alison Thornton, Russell Birkett

**Affiliations:** ^1^ Sheffield Children's NHS Foundation Trust Sheffield UK; ^2^ The University of Sheffield Sheffield UK

**Keywords:** catatonia, lorazepam, pediatric, psychiatry, psychosis

## Abstract

**Background:**

Catatonia is when someone does not respond to stimuli or their environment whilst awake. Catatonia can present in a variety of ways, including a change in movement, speech, and behavior. There is limited research on the causes and management of catatonia in an adult population and even more so in the pediatric population.

**Case Presentation:**

A 13‐year‐old presented with a 2‐week sudden onset history of distress and preoccupation regarding a child being in danger. Over the next few weeks, he was represented on multiple occasions and had a rapid weight loss due to refusing oral intake. He displayed increasing preoccupations and was admitted for intravenous fluids and commenced on nasogastric feeds. He declined rapidly, presenting with symptoms of catatonia, including stupor, catalepsy, mutism, and posturing. Physical health causes for this presentation were ruled out with imaging and bloods completed, and he was trialled on lorazepam. He responded well to the lorazepam, and after 5 days, the nasogastric tube was removed, and he commenced eating, drinking, communicating, and mobilizing. The lorazepam was reduced, and his symptoms reemerged. The lorazepam was titrated back up and reduced again at a slower rate.

**Conclusion:**

Whilst managing this case, it was noted that there is a lack of guidance available for catatonia in both adult and pediatric populations. There is particularly a lack of guidance on how to titrate onto and off lorazepam. This case highlighted a need for further research to support the development of guidelines for the management of pediatric catatonia.

## INTRODUCTION

Catatonia is when someone does not respond to stimuli or their environment whilst awake.^1^ Catatonia can present in a variety of ways, including a change in movement, speech, and behavior.^2^ It is a severe neuropsychiatric disorder and requires prompt recognition and treatment. It can be caused by a range of psychiatric conditions including psychosis, depression, obsessive compulsive disorder, and anxiety.^3^ In 20% of cases, there is an underlying organic cause, and so it is vital to rule out causes such as central nervous system lesions, infections, autoimmune conditions, and recent medication use.^4^

The management of catatonia in adults includes commencing lorazepam, and where there is no response, electroconvulsive therapy is considered.^5^ There is limited research on the causes and management of catatonia in an adult population and even more so in a pediatric population.

## CASE INFORMATION

A 13‐year‐old male presented to the accident and emergency department and was assessed by the child and adolescent mental health team.

### Initial presentation

The patient presented with a 2‐week sudden onset history of being highly anxious and preoccupied with thoughts about a child in danger in a neighboring flat. He reported he could hear screaming during the night and was actively trying to stay awake to look out of the windows. He also contacted the police on multiple occasions to report possible kidnappings.

On review, he was anxious and guarded, pacing and looking out of the windows, and expressing concerns about children. His speech was quiet, and responses were slow at times with limited content. There was no evidence of formal thought disorder and was not responding to unseen stimuli. He was admitted to an acute medical ward for further assessment.

### History of presenting complaint

The patient was not known to mental health services and had no past psychiatric history. There was no known family psychiatric history. Aside from talipes, his past medical history was unremarkable. The developmental history from his mother and the collateral history from education indicated a normal developmental pattern of social and communication milestones. The information from his educational setting identified him as a positive and well‐liked young person who transitioned well into secondary education. Motor coordination difficulties were identified in primary education and were assessed at that time, resulting in a diagnosis of dyspraxia. He was on the special education needs register at school due to this diagnosis; these difficulties did not significantly impact his overall functioning.

During our clinical assessments, no autistic traits were observed. A formal autism assessment was not considered during the acute phase of illness, as his presentation and developmental history did not suggest long‐standing social communication difficulties. In the future, if clinical concerns were to arise, a comprehensive neurodevelopmental assessment could be considered once his presentation has stabilized.

Research has documented that catatonia has been reported in pediatric patients with neurodevelopmental disorders, in particular autism and intellectual disability.^6^ To our knowledge, there are no specific studies that directly link dyspraxia with the risk of developing catatonia.

A month prior to the onset of this presentation, the patient reported feeling pressured by pupils at school to use a vape. It was subsequently disclosed that one pupil also convinced him he was an expert in dark magic and had cast black magic on him. The patient denied recreational drug use.

### Developments

The following day, he remained distressed and wanted to go home. His mother was concerned about his dietary intake. He reported he did not like the hospital food and was discharged home with community intensive treatment follow‐up.

Two days later, he was readmitted due to refusal of oral intake and a loss of 1.5 kg in 6 days. He reported he was fasting for Ramadan; his mother tried to reassure him that it was not Ramadan and did not need to fast. His mother reported that in the past 6 months, she had noted strange intense behaviors regarding his body image and dietary intake, such as fixating on developing a six‐pack, checking himself in the mirror, and a 6‐week period where he only ate chicken.

He was deemed medically fit with an outpatient referral to the eating disorders service due to the rapid weight loss. He was not accepted under the eating disorders team due to his presentation being predominantly anxiety‐related.

He lost a further 5.0 kg over 16 days and was readmitted; he met the criteria for eight red flags on the medical emergencies in eating disorders guidance.^7^ He presented with the following red flags: rapid weight loss, increased heart rate on standing, fluid refusal, severe dehydration, temperature less than 35.5°C, raised corrected QT interval (QTc) on the electrocardiogram (ECG), low white cell count, and acute food refusal.

He was started on intravenous fluids and declined the meal plan. He consented to nasogastric tube placement, and feeds commenced. He was advised to use a wheelchair to mobilize. Two days later, he refused feeds and was standing up for long periods up to 18 h a day, stating he needed to be ready to help the children. He began requesting to go home, stating he did not need to eat and that he needed to watch out for danger. A mental health assessment was conducted, and he was detained under Section 2 of the UK Mental Health Act.^8^ Section 2 is the compulsory detention for up to 28 days for the purpose of assessment of a mental disorder.^8^ On physical examination, he had bilateral pedal edema to mid‐calf, a low heart rate of 45 beats per minute, his bloods noted kidney dysfunction, and his ECG noted a raised QTc. He remained in an acute pediatric bed due to his physical health status.

### Management

Over the next few days, there was a rapid decline. He was sitting in his wheelchair, mute and holding his lips tightly shut, holding his saliva. There was no eye contact, no response to questions, and he had not passed urine for 24 h. He was presenting with symptoms of catatonia, including stupor, catalepsy, mutism, and posturing. Neuroimaging, including both computed tomography (CT) and magnetic resonance imaging (MRI) of the head, was within normal limits. His blood indicated low vitamin D, which was orally replaced. There is a lack of guidance available for the management of pediatric catatonia. The adult guidance on the management of catatonia was reviewed.^5^, ^9^ Due to the patient's age and weight, a clinical decision was made to commence lorazepam 1 mg three times daily and an additional 1 mg if needed; this was administered via the nasogastric tube.

The patient responded well to the lorazepam, and he began to communicate using pen and paper aids, continuing to display posturing on reviews. He reported that he was hearing voices and they were commanding him not to eat or talk. Five days into his treatment with lorazepam, he began eating, drinking, verbally communicating, and walking again, and his nasogastric tube was removed. He reported command auditory hallucinations, visual hallucinations, and facial illusion and distortions when looking in a mirror, as well as fixed and paranoid ideation and delusions about danger to children that he had to avert.

Due to the progress, we reviewed the lorazepam and discussed titrating this down. A review of the guidance indicated to reduce the lorazepam gradually,^3^, ^5^ did not quantify a recommended rate of reduction. We reduced the lorazepam to 1 mg twice per day in Week 1, and reduced it to 0.5 mg twice per day in Week 2. Following the second reduction, he became very concerned with children being in danger; he was not utilizing leave and began mobilizing less. A small number of patients will deteriorate in presentation following the reduction of lorazepam, and reinitiating is the advised management step in response to this.^10^

Catatonia guidance in adults advises to manage the symptoms of catatonia and to treat the underlying cause.^11^, ^12^ Benarous et al. highlighted the association of catatonia with psychiatric disorders in children and adolescents, in particular, schizophrenia.^13^ Sorg et al. noted the diagnostic and treatment challenges in a pediatric population.^14^ Both reviews noted that symptom management with the use of benzodiazepines and electroconvulsive therapy is the current evidence‐based treatment options.^13^, ^14^ Electroconvulsive therapy was not considered as a treatment option in this case, due to the response to benzodiazepines.

In this case, our formulation was catatonia secondary to a psychotic illness. This diagnosis was classified as 64A0 Catatonia associated with another mental disorder as per the International Classification of Diseases (ICD‐11).^15^ There was no affective component noted; he did not meet the criteria for a diagnosis of depression or mania. Schizophrenia or other primary psychotic disorders were considered in our differential diagnosis, given the history of functional decline, including not attending education, lack of sleep, and reduced oral intake. Alongside the functional decline, there was also the presence of auditory and visual hallucinations and delusional ideation. These features are consistent with an early onset of schizophrenia, in which catatonia was the initial presentation. Due to the initial presentation being a catatonic syndrome, this complicated early diagnostic clarity. The diagnosis of schizophrenia was not clear at the initial presentation, and the underlying psychotic symptoms only became apparent following the treatment of the catatonia with benzodiazepines. The collection of symptoms raises clinical suspicion of a psychotic disorder and represents a possible evolving schizophrenia. This case highlights the diagnostic challenges in pediatric catatonia and the importance of longitudinal follow‐up.

Due to the underlying psychotic symptoms following the improvement of the catatonic symptoms, an antipsychotic medication was commenced. A second‐generation antipsychotic was chosen, and after the review of benefits and side effect profile, aripiprazole was chosen.^9^ Aripiprazole was commenced at 2.5 mg daily, and lorazepam was increased back to 1 mg twice daily. In the UK Mental Health Act, Section 17 leave refers to authorized leave of absence from the hospital for detained patients. It is a temporary leave under specific conditions to support the progress and re‐integration of patients whilst under detention.^8^ As his presentation improved, he began to utilize Section 17 leave. At this time, Section 2 was due to expire; he was discharged from his section and was supported with the community intensive treatment team.

Aripiprazole was increased gradually to 7.5 mg, and the lorazepam was reduced at a slower rate, by 0.5 mg fortnightly until discontinued. Six weeks later, he was back to his baseline weight, returning to his hobbies, and planning his return to education. His care was then transferred to the community child and adolescent mental health team for ongoing monitoring.

## DISCUSSION

The diagnosis of catatonia in a pediatric population requires exploration of differential diagnoses and exclusion of organic causes. Neurological etiologies were evaluated due to the acute presentation of catatonic symptoms. A comprehensive neurological examination and neuroimaging, including both a CT and an MRI of the head, were conducted, and no abnormalities were found. A set of blood tests were also completed to rule out other potential causes, including infection, electrolyte disturbance, hepatic or renal causes, which were all within range. The potential of medication‐related symptoms was also considered with a full review of physical health history; there was no evidence of recent medications being prescribed. Neuroleptic malignant syndrome was ruled out due to the absence of fever, autonomic instability, rigidity, and elevated creatine kinase.^16^

Psychiatric causes were reviewed in this case, utilizing the mental state examinations, collateral information from family, and a longitudinal assessment. Psychiatric conditions were explored, including mood, anxiety, neurodevelopmental disorder, and trauma. The diagnosis of dyspraxia was considered in this context, and it may add to the complexity of this clinical presentation, in particular motor symptoms. However, there was no identified link with dyspraxia and the risk of catatonia in our research. In this case, the exclusion of alternative diagnoses supported the diagnosis of catatonia secondary to a psychotic disorder.^15^

The diagnosis of catatonia was made based upon the clinical symptoms and psychopathology in line with the ICD‐11 classification and the Royal College of Psychiatrists guidance.^2^, ^15^ A catatonia rating scale, such as the Pediatric Catatonia Rating Scale (PCRS) or Bush Francis Catatonia Rating Scale, was not used in this case.^17^, ^18^ Diagnosis and ongoing monitoring in this case were based upon multiple mental state examinations, observations, and reviewing the diagnostic criteria in the ICD‐11. In clinical practice, this approach is used in many settings; the use of a standardized rating tool may have provided quantification of catatonic symptoms and more precise monitoring of progress during treatment and follow‐up.

This case of pediatric catatonia highlighted the lack of guidance available for managing this condition. There are different suggestions and approaches recommended, but there is not a clear consensus on the dose, the length, and the rate of titration on and off Lorazepam. There is emerging evidence about benzodiazepine trials and the use of long‐term benzodiazepines in some patients.^9^, ^10^ The management approach in this case combined the adult guidance and clinical judgment of the individual.

There is limited pediatric guidance regarding catatonia, despite the incidence of catatonia in the pediatric population rising.^19^, ^20^ It is important that organic causes are considered in these presentations and that the underlying cause is sought to ensure the best treatment outcomes.

### Limitations

This case report has limitations that should be acknowledged. There was an absence of a standardized rating tool to aid in the diagnosis and monitoring of catatonia. The diagnosis and clinical monitoring were informed by multiple psychiatric assessments and diagnostic criteria; the absence of a rating tool limits symptom quantification and comparison over time. It highlights the value of incorporating rating tools in future case reports of pediatric catatonia.

The follow‐up period in this case was limited; this prevented a longitudinal assessment of symptoms and a review of how the symptoms progressed over time. The case report covered the time spent within the community intensive team; the patient was then handed over to the community team for ongoing support. Over time, there may have been a consideration of an alternative diagnosis or clarity provided. This case highlights the diagnostic challenges in pediatric catatonic and its underlying conditions.

A range of differential diagnoses were considered; however, there were some investigations that should have been conducted in a full assessment of catatonia. In particular, investigations such as electroencephalography (EEG) and a lumbar puncture to analyze cerebrospinal fluid were not performed. These investigations are recommended to aid thorough neurological evaluation.^9^ Additionally, drug toxicology screening was not completed, limiting the ability to exclude substance use in this case.

This report highlights the importance of research and guidance into pediatric catatonia and the diagnostic complexity in this population. This case emphasizes the importance of comprehensive assessments and longitudinal follow‐up in similar cases.

## CONCLUSION

There is a need for further research to support the development of guidelines for the management approach for pediatric catatonia. The incidence of catatonia is rising, and clear guidance on the medical approaches is vital to ensure the best outcomes for the patient's journey. The authors also note that whilst medical management was effective in the treatment of symptoms, the importance of non‐medical management should not be overlooked. Non‐medical management such as shared decision‐making with families and building a routine for the patient during their recovery, has a positive impact on the patient journey.

## AUTHOR CONTRIBUTIONS

**Alison Thornton**: Conceptualization; writing—draft preparation; writing—reviewing and editing; visualization; methodology. **Russell Birkett**: Supervision; conceptualization; writing—reviewing and editing; visualization, methodology.

## CONFLICT OF INTEREST STATEMENT

The authors declare no conflicts of interest.

## ETHICS APPROVAL STATEMENT

N/A.

## PATIENT CONSENT STATEMENT

Verbal and written consent was provided by the patient to complete this case report.

## CLINICAL TRIAL REGISTRATION

N/A.

## Data Availability

The data that support the findings of this study are available from the corresponding author upon reasonable request.

## References

[1] Barnes MP , Saunders M , Walls TJ , Saunders I , Kirk CA . The syndrome of Karl Ludwig Kahlbaum. J Neurol Neurosurg Psychiatry. 1986;49(9):991–996. 3760905 10.1136/jnnp.49.9.991 PMC1029001

[2] Catatonia Royal College of Psychiatrists [Internet]. 2022 [cited 2025 Aug 30]. Available from: https://www.rcpsych.ac.uk/mental-health/mental-illnesses-and-mental-health-problems/catatonia

[3] Catatonia|UCL Faculty of Brain Sciences [Internet]. 2024 [cited 2025 Aug 30]. Available from: https://www.ucl.ac.uk/brain-sciences/psychiatry/our-research/catatonia

[4] Rogers JP , Pollak TA , Blackman G , David AS . Catatonia and the immune system: a review. Lancet Psychiatry. 2019 Jul 1;6(7):620–630. Available from: https://www.sciencedirect.com/science/article/pii/S2215036619301907?via%3Dihub 31196793 10.1016/S2215-0366(19)30190-7 PMC7185541

[5] Taylor D , Barnes TE , Young A . The Maudsley prescribing guidelines in psychiatry. 13th ed. 2013.

[6] Hauptman AJ , Cohen D , Dhossche D , Raffin M , Wachtel L , Ferrafiat V . Catatonia in neurodevelopmental disorders: assessing catatonic deterioration from baseline. Lancet Psychiatry. 2023;10:228–234. 36708735 10.1016/S2215-0366(22)00436-9

[7] CR233: Medical Emergencies in Eating Disorders: Guidance on Recognition and Management [Internet]. 2022. Available from: https://www.rcpsych.ac.uk/docs/default-source/improving-care/better-mh-policy/college-reports/college-report-cr233-medical-emergencies-in-eating-disorders-(meed)-guidance.pdf?sfvrsn=2d327483_63

[8] McLoughlin IJ . Detention under the Mental Health Act. Psychiatr Bull R Coll Psychiatr. 1995;19(8):514–515.

[9] Rogers JP , Oldham MA , Fricchione G , Northoff G , Ellen Wilson J , Mann SC , et al. Evidence‐based consensus guidelines for the management of catatonia: recommendations from the British Association for Psychopharmacology. J Psychopharmacol. 2023 Apr 1;37(4):327–369. Available from: https://pmc.ncbi.nlm.nih.gov/articles/PMC10101189/ 37039129 10.1177/02698811231158232 PMC10101189

[10] Ali SF , Gowda GS , Jaisoorya TS , Math SB . Resurgence of catatonia following tapering or stoppage of lorazepam—a case series and implications. Asian J Psychiatry. 2017 Aug 1;28:102–105. Available from: https://pubmed.ncbi.nlm.nih.gov/28784360/ 10.1016/j.ajp.2017.04.002 28784360

[11] Seethalakshmi R , Dhavale S , Suggu K , Dewan M . Catatonic syndrome: importance of detection and treatment with lorazepam. Ann Clin Psychiatry. 2008 Feb;20(1):5–8. 18297580 10.1080/10401230701844786

[12] Hirjak D , Rogers JP , Wolf RC , Kubera KM , Fritze S , Wilson JE , et al. Catatonia. Nat Rev Dis Primers. 2024 Dec 1;10(1):49–50. 39025858 10.1038/s41572-024-00534-w

[13] Benarous X , Raffin M , Ferrafiat V , Consoli A , Cohen D . Catatonia in children and adolescents: new perspectives. Schizophr Res. 2018;200:56–67. 28754582 10.1016/j.schres.2017.07.028

[14] Sorg EM , Chaney‐Catchpole M , Hazen EP . Pediatric catatonia: a case series‐based review of presentation, evaluation, and management. Psychosomatics. 2018;59:531–538. 30104020 10.1016/j.psym.2018.05.012

[15] World Health Organization . International Classification of Diseases, 11th Revision (ICD‐11). Geneva: World Health Organization; 2022.

[16] Brown AE , Giritharan A , Phibbs FT . Neuroleptic malignant syndrome. In: Primer on the autonomic nervous system. 4th ed. 2022.

[17] Benarous X , Consoli A , Raffin M , Bodeau N , Giannitelli M , Cohen D , et al. Validation of the Pediatric Catatonia Rating Scale (PCRS). Schizophr Res. 2016;176(2–3):378–386. 27377978 10.1016/j.schres.2016.06.020

[18] Aandi Subramaniyam B , Muliyala KP , Suchandra HH , Reddi VSK . Diagnosing catatonia and its dimensions: cluster analysis and factor solution using the Bush Francis Catatonia Rating Scale (BFCRS). Asian J Psychiatry. 2020;52:102002. 10.1016/j.ajp.2020.102002 32506001

[19] Soe KC , Arnold MJ , Smith JR , Dominick KC . Is pediatric catatonia on the rise? Hosp Pediatr. 2025 Sep 1;15(9):e449–e452. 10.1542/hpeds.2025-008504 40819834 PMC12439331

[20] Lichtor SA , Dunn E , Ray A , Wulff C , Anglero‐Diaz Y , O'Connor R , et al. Rising incidence of pediatric catatonia and medical etiology: multiyear trends. Hosp Pediatr. 2025 Sep 1;15(9):711–720. Available from: https://publications.aap.org/hospitalpediatrics/article/15/9/711/203186/Rising-Incidence-of-Pediatric-Catatonia-and 40819835 10.1542/hpeds.2025-008373

